# Prognostic value of abdominal aortic calcification in patients with esophageal cancer undergoing neoadjuvant therapy and esophagectomy

**DOI:** 10.1007/s00261-026-05511-3

**Published:** 2026-04-21

**Authors:** Jacob Jalil Hassan, Daniele Romeo, Stefan Niebisch, Matthias Mehdorn, Daniel Seehofer, Gertraud Stocker, Florian Lordick, Timm Denecke, Hans-Jonas Meyer

**Affiliations:** 1 Department of Diagnostic and Interventional Radiology, University Hospital Leipzig, Leipzig, Germany; 2 Department of Biomedicine, Neuroscience and Advanced Diagnostics (BiND), University of Palermo, Palermo, Italy; 3 Department of Visceral and Transplantation Surgery, University Hospital Leipzig, University of Leipzig, Leipzig, Germany; 4 Department of Oncology, Gastroenterology, Hepatology, and Pulmonology, University of Leipzig Medical Center, Leipzig, Germany

**Keywords:** Esophageal cancer, CT, Aortic calcification, Prognosis, Neoadjuvant therapy

## Abstract

**Background:**

Computed tomography (CT) is a first line imaging tool for staging of esophageal cancer (EC). Previous studies have shown promising results of the prognostic relevance of vessel calcifications quantified by CT, especially coronary and aortic calcifications, in oncological patients. The aim of this study was to analyze the prognostic relevance of aortic calcification assessed in staging CT in patients with EC undergoing curative treatment.

**Methods:**

All patients with EC treated with neoadjuvant therapy followed by curative resection at the University of Leipzig Medical Center, a tertiary care hospital, were retrospectively evaluated between 2016 and 2023. A total of 89 patients were included in the analysis. Abdominal aorta and iliac artery calcification volume was measured in a semi-automated fashion at baseline before neoadjuvant therapy using staging CT images. The primary endpoint was overall survival and disease-free survival was assessed as a secondary endpoint. For statistical analysis group differences were calculated using the Mann-Whitney-U test. Kaplan-Meier curves and multivariable Cox regression analysis were used to test the effect of aortic calcification volume and clinical variables on mortality.

**Results:**

In univariable Cox regression, higher abdominal aortic calcification volume was significantly associated with shorter overall survival (Hazard ratio (HR) 1.259 per 1 cm³, 95% CI: 1.100–1.411, *p* < 0.001) and disease-free survival (HR 1.279 per 1 cm³, 95% CI: 1.135–1.411, *p* < 0.001). The abdominal aortic calcification volume remained an independent predictor of overall survival in multivariable analysis (HR 1.248, 95% CI 1.069–1.458, *p* = 0.005). Kaplan–Meier analyses demonstrated progressively reduced overall survival and disease-free survival with increasing abdominal aortic calcification volume.

**Conclusions:**

CT-quantified abdominal aortic calcification provides independent prognostic information for both overall and disease-free survival in patients with EC undergoing neoadjuvant therapy followed by curative resection. Prospective evaluations of CT-defined vessel calcification and potential translation in clinical routine seem promising.

**Supplementary Information:**

The online version contains supplementary material available at https://doi.org/10.1007/s00261-026-05511-3.

## Introduction

Esophageal cancer (EC) ranks among the most prevalent malignancies and is the sixth leading cause of cancer-related mortality worldwide [1]. Although diagnostic and therapeutic strategies have advanced, the 5-year survival rate remains poor at approximately 15–20% [2]. In patients with locally advanced disease, the current standard treatment is neoadjuvant therapy followed by curative surgical resection, which offers superior survival compared to surgery alone [3, 4]. Nevertheless, prognosis varies considerably among individuals, which is not fully captured by the conventional TNM (Tumor–Node–Metastasis) classification. This heterogeneity highlights the need for novel prognostic biomarkers to refine risk stratification.

Traditionally, tumor-centric factors such as TNM-stage and histopathological features have been used as the primary determinants of prognosis in EC. However, there is growing recognition that patient-related factors and comorbidities also substantially influence oncological outcomes [5, 6]. In particular, cardiovascular health and systemic atherosclerosis may play an underappreciated role in cancer prognosis [7, 8]. Many patients with EC are older, have a history of smoking and suffer from obesity, all of which are established risk factors that predispose to atherosclerosis [9]. Vascular calcification is readily visible on routine computed tomography (CT) imaging [10]. It is plausible that an elevated burden of vascular calcification could serve as an imaging biomarker reflecting an individual’s overall health status or frailty, which in turn might impact their ability to tolerate cancer treatments or recover from surgery [11]. In this context, measuring aortic calcification on preoperative scans could provide additional prognostic information beyond standard oncologic staging.

Indeed, recent evidence suggests that vascular calcification has prognostic significance across various malignancies. A recent meta-analysis on incidental coronary artery calcification (CAC) in lung cancer patients reported a pooled hazard ratio of 1.56 for mortality in patients with substantial CAC [12]. A comparable association has been observed in gastrointestinal oncology, where gastric cancer patients who underwent curative gastrectomy and had high abdominal aortic calcification volume (AACV) experienced significantly shorter survival [13]. In the setting of EC, atherosclerotic calcification of the aorta has been identified as an independent risk factor for anastomotic leakage after esophagectomy [14, 15]. However, no study to date has evaluated whether AACV provides prognostic information for overall survival in esophageal cancer, leaving it unclear whether it adds value beyond conventional TNM staging and clinical factors. This constitutes an important gap, as AACV is easily quantifiable on routine staging CT and may serve as a simple imaging biomarker to improve prognostic stratification.

Therefore, the present study elucidates the prognostic relevance of aortic calcification quantified by CT images in patients with EC treated with neoadjuvant therapy and esophagectomy. The study hypothesizes that greater AACV could be associated with shorter overall survival, independent of established prognostic factors and age.

## Methods

### Inclusion and exclusion criteria

Patients treated for locally advanced esophageal cancer at our tertiary care center between 2016 and 2023 were retrospectively screened for eligibility. Inclusion criteria were histologically confirmed esophageal cancer, treatment with neoadjuvant therapy followed by curative-intent esophagectomy at our institution, and availability of a non-contrast-enhanced CT scan performed within 12 months before surgery. Patients were excluded if essential imaging or clinical data were missing, if CT image quality was insufficient for reliable calcification quantification, or if they had received definitive radiochemotherapy instead of surgery.

## Study design and data collection

Neoadjuvant treatment consisted of either chemoradiotherapy according to the CROSS (Chemoradiotherapy for Oesophageal Cancer followed by Surgery Study) protocol or chemotherapy according to the FLOT (5-Fluorouracil, Leucovorin, Oxaliplatin, Docetaxel) regimen. At our institution, the choice between the CROSS and FLOT regimens was based on multidisciplinary tumor board recommendations and prevailing clinical guidelines, with CROSS more commonly applied in squamous cell carcinoma and FLOT more frequently used in adenocarcinoma. Demographic and tumor-related characteristics, including age, sex, histological subtype, TNM-stage and tumor grade were obtained from medical records. The primary endpoint was to evaluate the prognostic value of CT-based vascular calcification of the aorta for overall survival in patients with locally advanced esophageal carcinoma undergoing neoadjuvant therapy followed by curative resection. Disease-free survival was assessed as a secondary endpoint.

## Vascular calcification quantification

**Fig. 1:**
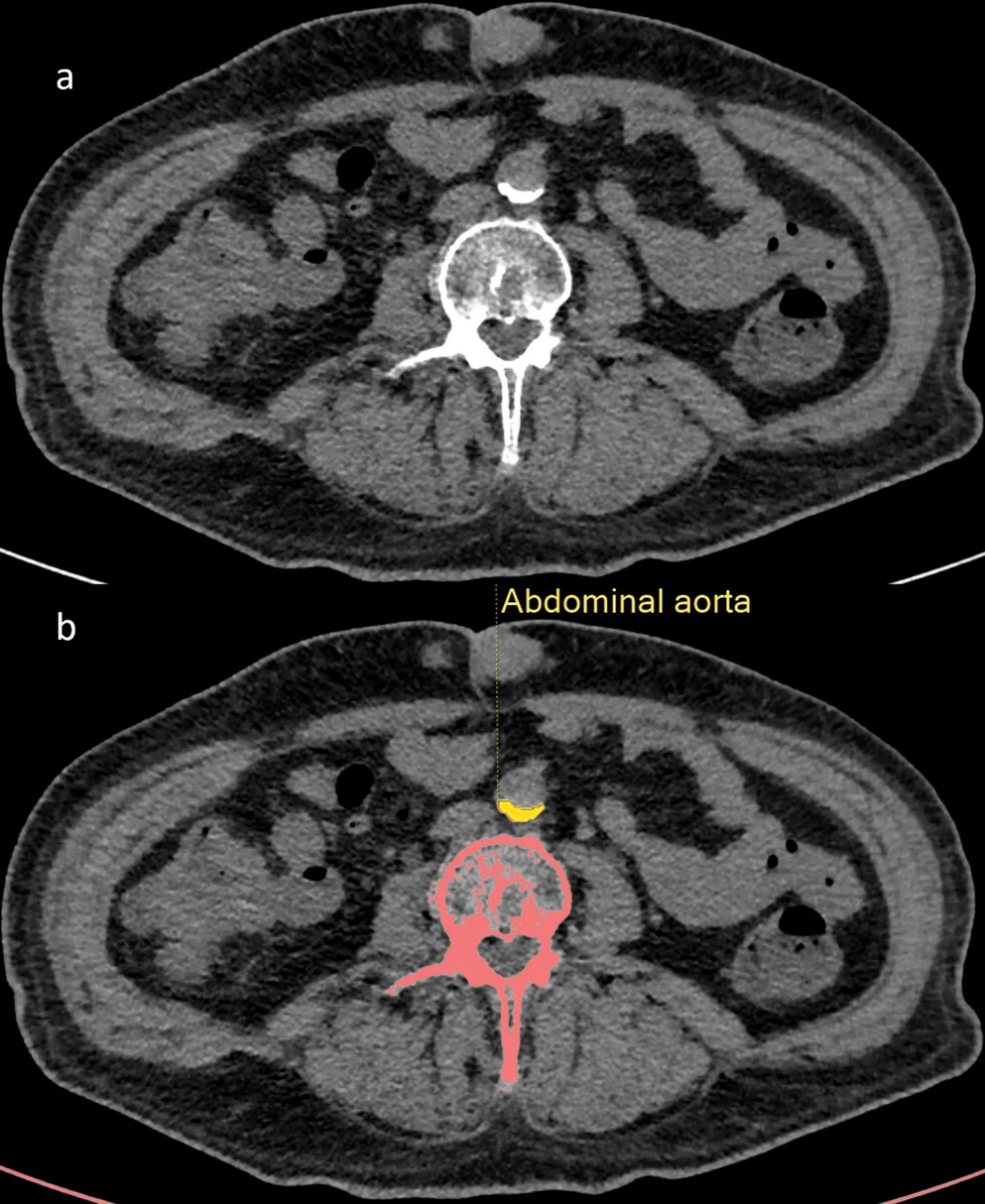
Representative axial non-contrast-enhanced CT image of a patient with esophageal cancer illustrating semi-automated abdominal aortic calcification scoring. **a** Original CT slice. **b** Segmentation result with calcified plaque in the abdominal aorta highlighted in yellow. Other dense structures, such as the vertebral body, which exceed the predefined threshold of 130 Hounsfield units, are highlighted in red

The non-contrast-enhanced CT images used for calcification quantification were derived from the CT component of routine PET/CT examinations performed using multidetector scanners with a slice thickness of ≤ 5 mm. Digital Imaging and Communications in Medicine **(**DICOM) data were retrieved from the institutional Picture Archiving and Communication System (PACS) for volumetric analysis. Vascular calcifications were quantified semi-automatically using Philips Advanced Visualization Workspace (Philips Healthcare, Amsterdam, the Netherlands) with manual corrections performed when required. The advanced vessel tool was used for the calculations. Regions of interest (ROIs) were defined from the lower renal artery origin to the aortic bifurcation for the infrarenal aorta and from the aortic bifurcation to the iliac bifurcation for the common iliac arteries. A threshold of > 130 Hounsfield units was applied to identify calcified voxels. Calcification volumes were measured in cubic centimetres (cm³) for each vascular segment, and the total calcification burden was calculated as the sum of infrarenal aortic and bilateral common iliac artery volumes. Interobserver reliability was assessed based on independent measurements performed by two readers in a subset of 20 patients using a two-way mixed-effects model. A representative image of the calcium scoring is shown in Fig. 1.

### Statistical analysis

All statistical analyses were performed using IBM SPSS Statistics (version 31.0.0.0, IBM Corp., Armonk, NY, USA). Continuous variables are presented as medians with interquartile ranges (IQR) and categorical variables as counts and percentages. Group differences were calculated using the Mann-Whitney-U test.

Overall survival (OS) was defined as the time from the date of surgery to death from any cause or last follow-up. Disease-free survival (DFS) was defined as the time from the date of surgery to the first documented tumor recurrence or death, whichever occurred first. Patients without an event were censored at the date of last follow-up.

Univariable and multivariable Cox proportional hazards regression models were used to assess the association between calcification volume and overall survival, reporting hazard ratios (HR) with confidence intervals (CI). For regression analysis, the ordinal variable T-stage was dichotomized into low (T0-2) and high (T3/4) categories. Likewise, age was dichotomized at 65 years (< 65 vs. ≥65 years). Variables with *p* < 0.1 in univariable analysis were included in the multivariable model. Abdominal aortic calcification volume (AACV), iliac artery calcification volume (IACV), and combined abdominal aortic and iliac artery calcification volume (AAIACV) were each analyzed in univariable Cox regression. As AACV demonstrated the strongest association with overall survival, only AACV was included in the multivariable analysis to avoid collinearity between calcification measures. To assess potential non-linear associations between AACV and survival outcomes, restricted cubic spline analyses with four knots placed at the 5th, 35th, 65th, and 95th percentiles of the AACV distribution were performed within Cox proportional hazards models for OS and DFS. A likelihood ratio test comparing the spline model with the corresponding linear model was used to evaluate deviation from linearity. Kaplan–Meier survival curves were compared using the log-rank test. A two-sided *p* < 0.05 was considered statistically significant.

## Results

### Patient characteristics

A total of 89 patients were included in the analysis. During follow-up, 32 patients died, and 47 events were recorded for disease-free survival (death or recurrence). The median age at diagnosis was 61 years (interquartile range [IQR] 54–70). Most patients were male (*n* = 77, 86.5%). The median time interval between the pretherapeutic CT scan and surgery was 106 days (IQR: 95–133). In all cases the CT was performed prior to the neoadjuvant therapy.

Adenocarcinoma was the predominant histological subtype (*n* = 60, 67.4%), followed by squamous cell carcinoma (*n* = 29, 32.6). T3 was the most frequent T-stage (*n* = 61, 68.5%). Nodal involvement (N1-3) was observed in 69 patients (77.5%) and distant metastases were absent in all cases. G2 and G3 were the most common tumor grades (G2: *n* = 38, 42.7% - G3: *n* = 33, 37.1%). The median AACV was 1.007 cm³ (IQR: 0.253–3.552) and 0.755 cm³ (IQR: 0.188–1.928) for the IACV. The combined median calcification volume of the AAIACV was 1.816 cm³ (IQR: 0.503–5.890). Interobserver agreement for abdominal aortic calcification volume measurements demonstrated good reliability with an intraclass correlation coefficient (ICC) of 0.762 (95% CI: 0.439–0.882). The median follow-up of survivors was 1,141 days (37.5 months). Baseline patient characteristics are summarized in Table 1.

**Table 1:** Patient characteristics of the investigated cohort (*n* = 89)

Patient characteristics	Median (IQR) or *n* (%)
Age	61 years (IQR 54–70)
Male	77 (86.5%)
Female	12 (13.5%)
Interval between CT and surgery	106 days (IQR 95–133)
Squamous cell carcinoma	29 (32.6%)
Adenocarcinoma	60 (67.4%)
T0	4 (4.5%)
T1	6 (6.7%)
T2	13 (14.6%)
T3	61 (68.5%)
T4	5 (5.6%)
N0	20 (22.5%)
N1-3	69 (77.5%)
M0	89 (100%)
G1	10 (11.2%)
G2	38 (42.7%)
G3	33 (37.1%)
AACV	1.007 cm^3^ (IQR 0.253–3.552)
IACV	0.755 cm^3^ (IQR 0.188–1.928)
AAIACV	1.816 cm^3^ (IQR 0.503–5.89)

Tumor grading data were missing in 8 cases (*n* = 81). Abbreviations: abdominal aortic calcification volume (AACV); iliac artery calcification volume (IACV); combined abdominal aortic and iliac artery calcification volume (AAIACV); tumor stage (T); nodal stage (N); metastasis stage (M); tumor grade (G)

## Univariable cox regression analysis overall survival

In univariable Cox regression, higher AACV was significantly associated with shorter overall survival (OS) (HR 1.259 per 1 cm³, 95% CI: 1.100–1.411, *p* < 0.001). Higher IACV was also significantly associated with shorter OS (HR 1.206 per 1 cm³, 95% CI: 1.031–1.411, *p* = 0.019). Likewise, higher total calcification volume (AAIACV) was significantly associated with shorter OS (HR 1.121 per 1 cm³, 95% CI: 1.041–1.207, *p* = 0.003). Among these measures, AACV alone demonstrated the strongest association with mortality. Therefore, all subsequent analyses were restricted to AACV.

Among clinical variables age (> 65 years) was significantly associated with worse OS (HR 2.444, 95% CI 1.192–5.011, *p* = 0.015). In contrast, sex (HR 1.036, *p* = 0.948), T-status (HR 1.129, *p* = 0.778), N-status (HR 1.250, *p* = 0.623), tumor grading (HR 1.801, *p* = 0.058) and histologic subtype (HR 0.898, *p* = 0.779) were not significantly associated with OS.

**Fig. 2:**
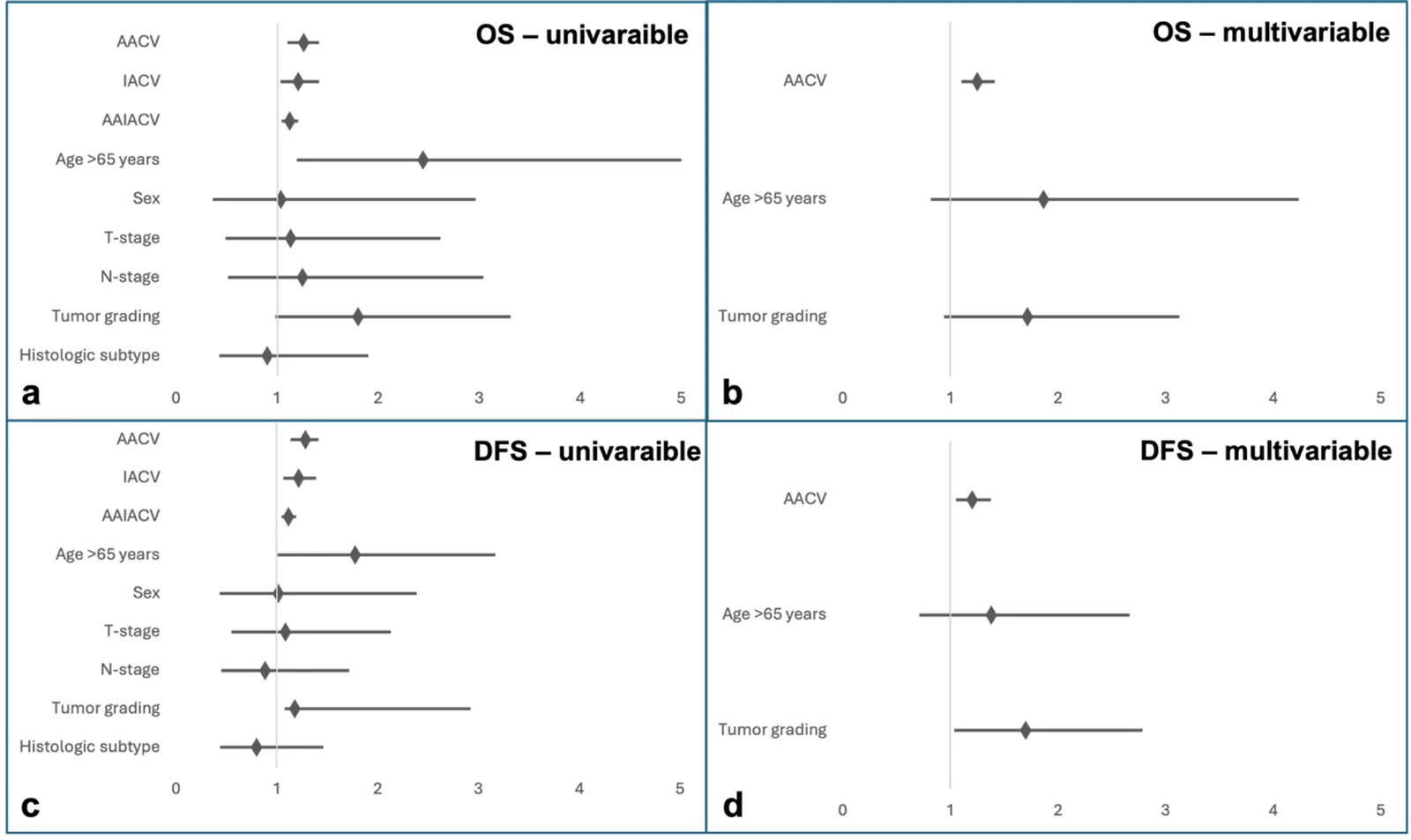
Forest plots showing hazard ratios (HR) with 95% confidence intervals (CI) from univariable and multivariable Cox regression analyses. **a** Shows the full univariable model including calcification measures and clinical variables for overall survival (OS). **b** Shows the variables included in the multivariable model for OS. **c** Shows the full univariable model including calcification measures and clinical variables for disease-free survival (DFS). **d** Shows the variables included in the multivariable model for DFS

The results of the Cox regression analyses are shown in Table 2, with the corresponding forest plots presented in Fig. 2.

**Table 2:** Univariate and multivariable Cox regression analyses for overall survival

	Univariable analysis		Multivariable analysis	
	HR (95 CI)	*p*-value	HR (95 CI)	*p*-value
AACV	1.259 (1.100–1.411)	< 0.001	1.248 (1.069–1.458)	0.005
IACV	1.206 (1.031–1.411)	0.019		
AAIACV	1.121 (1.041–1.207)	0.003		
Age > 65 years	2.444 (1.192–5.011)	0.015	1.862 (0.818–4.237)	0.138
Sex	1.036 (0.362–2.966)	0.948		
T-stage	1.129 (0.487–2.616)	0.778		
N-stage	1.250 (0.514–3.040)	0.623		
Tumor grading	1.801 (0.981–3.307)	0.058	1.715 (0.94–3.128)	0.079
Histologic subtype	0.898 (0.425–1.900)	0.779		

Hazard ratios (HR) with 95% confidence intervals (CI) and p-values are shown for each variable. Abbreviations: abdominal aortic calcification volume (AACV); iliac artery calcification volume (IACV); combined abdominal aortic and iliac artery calcification volume (AAIACV), tumor stage (T-stage); nodal stage (N-stage)

## Multivariable cox regression analysis overall survival

Multivariable Cox regression including AACV, age and tumor grading demonstrated AACV as the only independent predictor of shorter overall survival (HR 1.248, 95% CI 1.069–1.458, *p* = 0.005). Age and tumor grading were not significantly associated with overall survival in the multivariable model.

### Univariable cox regression analysis disease-free survival

In univariable Cox regression, higher AACV was significantly associated with shorter disease-free survival (DFS) (*p* < 0.001). Higher IACV was also significantly associated with shorter DFS (HR 1.214 per 1 cm³, 95% CI: 1.063–1.385, *p* = 0.004). Likewise, AAIACV was significantly associated with shorter DFS (HR 1.114 per 1 cm³, 95% CI: 1.046–1.188, *p* < 0.001). Among clinical variables, tumor grading was significantly associated with worse DFS (HR 1.772, 95% CI: 1.075–2.921, *p* = 0.025). In contrast, age (HR 1.774, *p* = 0.052), sex (HR 1.010, *p* = 0.981), T-status (HR 1.081, *p* = 0.822), N-status (HR 0.881, *p* = 0.712), and histologic subtype (HR 0.797, *p* = 0.461) were not significantly associated with DFS (Table 3).

**Table 3:** Univariate and multivariable Cox regression analyses for disease free survival

	Univariable analysis		Multivariable analysis	
	HR (95 CI)	*p*-value	HR (95 CI)	*p*-value
AACV	1.279 (1.135–1.411)	< 0.001	1.202 (1.051–1.374)	0.007
IACV	1.214 (1.063–1.385)	0.004		
AAIACV	1.114 (1.046–1.188)	< 0.001		
Age > 65 years	1.774 (0.996–3.163)	0.052	1.378 (0.712–2.666)	0.341
Sex	1.010 (0.428–2.386)	0.981		
T-stage	1.081 (0.549–2.126)	0.822		
N-stage	0.881 (0.448–1.731)	0.712		
Tumor grading	1.772 (1.075–2.921)	0.025	1.696 (1.033–2.784)	0.037
Histologic subtype	0.797 (0.435–1.458)	0.461		

Hazard ratios (HR) with 95% confidence intervals (CI) and p-values are shown for each variable. Abbreviations: abdominal aortic calcification volume (AACV); iliac artery calcification volume (IACV); combined abdominal aortic and iliac artery calcification volume (AAIACV), tumor stage (T-stage); nodal stage (N-stage)

### Multivariable cox regression analysis disease-free survival

Multivariable Cox regression including AACV, tumor grading and age demonstrated an independent association of AACV with shorter DFS (HR 1.202, 95% CI: 1.051–1.374, *p* = 0.007). Tumor grading also remained significantly associated with DFS (HR 1.696, 95% CI: 1.033–2.784, *p* = 0.037), whereas age (> 65 years) was not independently associated with DFS (HR 1.378, 95% CI: 0.712–2.666, *p* = 0.341).

### Non-linear association analysis

Restricted cubic spline analysis did not indicate evidence of a non-linear association between AACV and overall survival (*p* = 0.61) or disease-free survival (*p* = 0.67). The corresponding spline curves are shown in Supplementary Material 1.

### Kaplan–Meier survival analysis overall survival

**Fig. 3:**
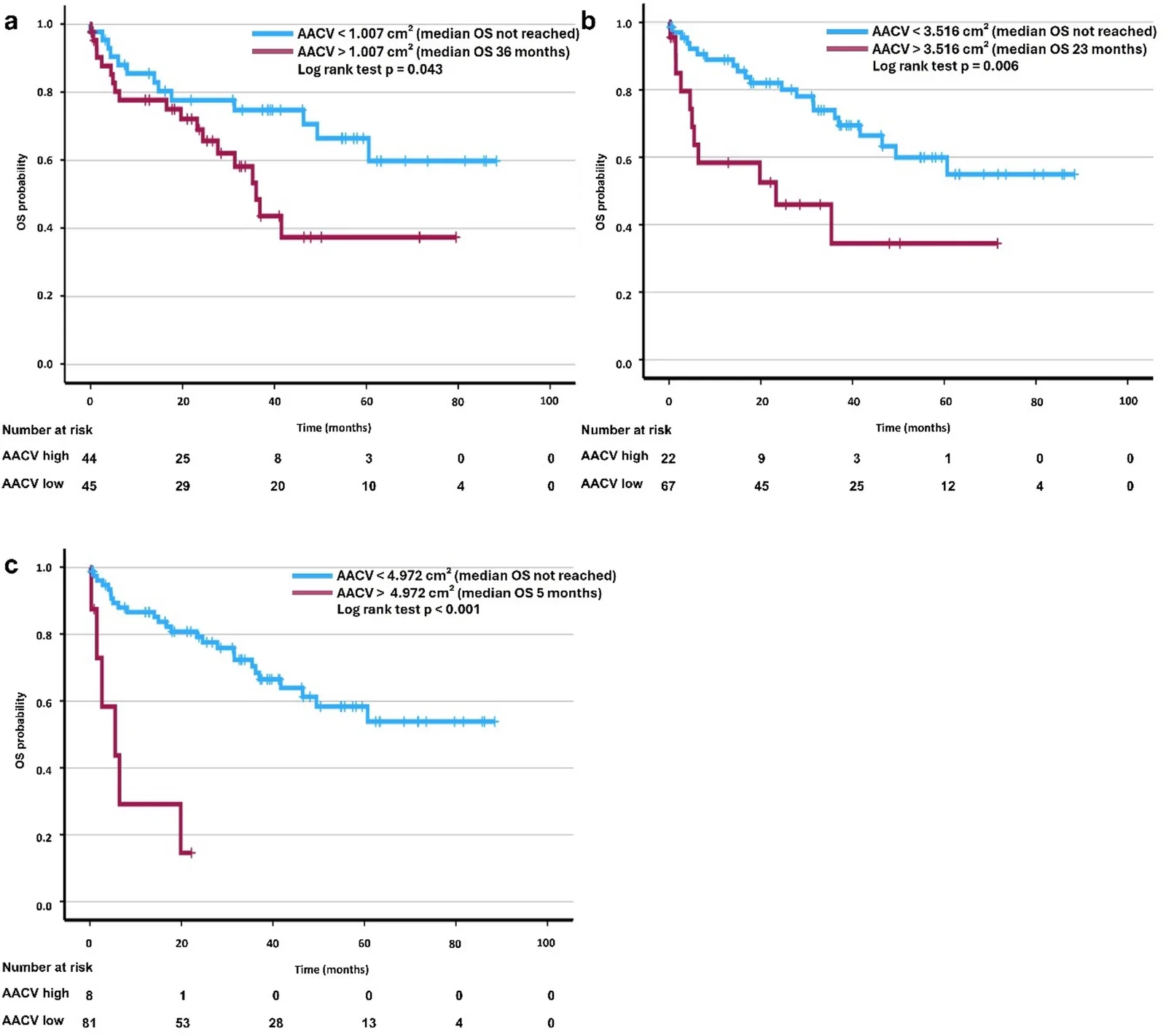
Kaplan–Meier overall survival (OS) curves stratified by abdominal aortic calcification volume (AACV). Patients were divided according to the median (**a**), the upper quartile (**b**), and the upper 10% cut-off of AACV (**c**). Numbers at risk at 0, 20, 40, 60, 80, and 100 months are displayed below each plot. In subgroups with lower AACV, the median overall survival was not reached

Kaplan–Meier analysis demonstrated a stepwise association between higher AACV and reduced OS. When dichotomized at the median AACV (1.007 cm³), patients with higher AACV showed a significantly shorter OS compared to those below the median (median OS not reached vs. 36 months, *p* = 0.043). Using the upper quartile threshold (3.516 cm³), patients with higher AACV exhibited an even more pronounced survival disadvantage (median OS not reached vs. 23 months, *p* = 0.006). This effect was strongest when applying the upper 10% threshold (4.972 cm³), where patients with high AACV showed markedly reduced OS (median OS not reached vs. 5 months, *p* < 0.001). In all analyses, the median OS was not reached in the groups with lower AACV. The Kaplan-Meier survival curves are shown in Fig. 3.

### Kaplan–Meier survival analysis disease-free survival

**Fig. 4:**
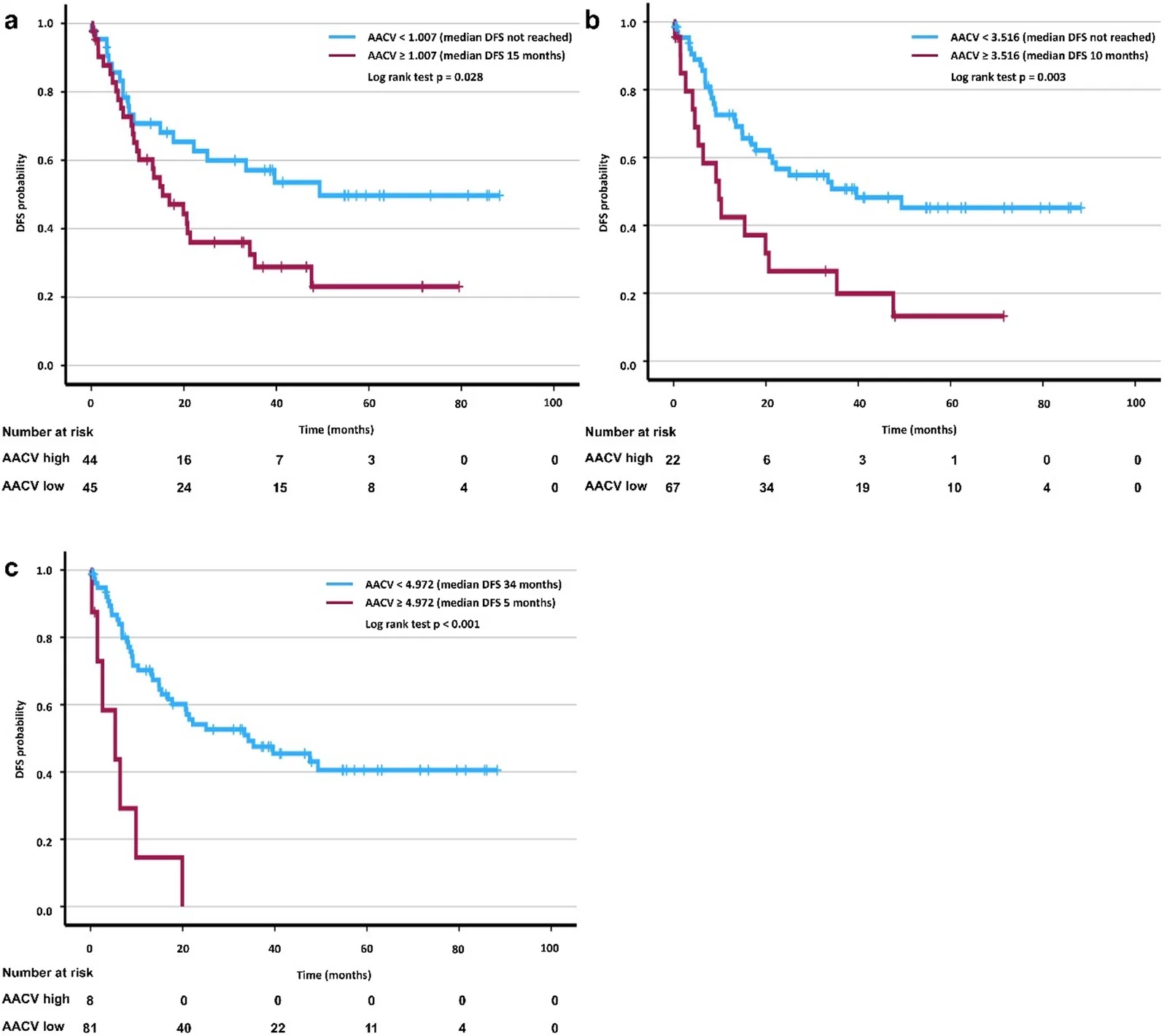
Kaplan–Meier disease-free survival (DFS) curves stratified by abdominal aortic calcification volume (AACV). Patients were divided according to the median (**a**), the upper quartile (**a**), and the upper 10% cut-off of AACV (**a**). Numbers at risk at 0, 20, 40, 60, 80, and 100 months are displayed below each plot

Kaplan–Meier analysis demonstrated a stepwise association between higher AACV and reduced DFS. When dichotomized at the median AACV (1.007 cm³), patients with higher AACV showed a significantly shorter DFS compared with those below the median (median DFS not reached vs. 15 months, *p* = 0.028). Using the upper quartile threshold, DFS separation became more pronounced (median DFS not reached vs. 10 months, *p* = 0.003). This effect was strongest when applying the upper 10% threshold, where patients with very high AACV exhibited markedly reduced DFS (median DFS: 34 months vs. 5 months, log-rank *p* < 0.001). The Kaplan-Meier survival curves are shown in Fig. 4.

## Discussion

This study demonstrates that CT-derived AACV is an independent prognostic imaging marker for overall survival (OS) and disease-free survival (DFS) in patients with esophageal cancer undergoing neoadjuvant therapy and curative resection. The prognostic effect remained significant after adjustment for covariables underscoring that vascular calcification provides additional risk information beyond conventional clinical and histological tumor staging. While AACV, IACV, and AAIACV were each associated with OS and DFS in univariable analysis, AACV showed the largest effect size and was therefore prioritized for multivariable modelling with subsequent analyses focusing on AACV to avoid redundancy between related calcification measures.

The current study is in line with recent exploratory research highlighting the overall condition of tumor patients defined by quantitative features derived from staging CT images. While body composition parameters such as sarcopenia and visceral adiposity have been associated with adverse outcomes in esophageal cancer, vascular calcification has received comparatively little attention. A large meta-analysis including 5,965 patients reported that sarcopenia is associated with worse OS, with a pooled HR of 1.68 (CI: 1.54–1.83) [16].

Although vascular calcification has been described as a predictor of postoperative morbidity, its prognostic value for overall survival in esophageal cancer has not been shown until now. Prior studies have demonstrated that higher thoracic aortic or celiac trunk calcification increases the risk of anastomotic leakage following esophagectomy [14, 15, 17]. Our results support the hypothesis that the vascular calcification reflects a broader systemic vulnerability, contributing to reduced survival independent of tumor-intrinsic features. Each increment in AACV was associated with a substantially higher hazard of mortality, underscoring the clinical relevance of this simple to measure imaging feature.

The prognostic effect of vascular calcification has also been described in other malignancies. Coronary artery calcification has been associated with worse outcomes in lung cancer [12]. Furthermore, it has been shown that coronary artery calcification is moderately correlated with calcification of the aorta, indicating a link between aortic calcification and cardiovascular frailty [18]. A recent study with a similar study design to ours, showed that AACV predicted OS in patients with gastric cancer who underwent curative gastrectomy [13]. In that study, the calcification volume was quantified from the infrarenal aorta to the iliac bifurcation. In our cohort, infrarenal aortic calcification alone showed greater prognostic value than calcification of the common iliac arteries or the combined infrarenal aortic and iliac calcification volume.

Interestingly, aortic calcification may not be static. Evidence from non-small cell lung cancer suggests that chemoradiotherapy can accelerate aortic calcification [19]. It is conceivable that patients who accumulate greater vascular calcification during neoadjuvant therapy are particularly susceptible to postoperative complications and inferior long-term outcomes. Future longitudinal studies assessing vascular changes during oncologic therapy may help clarify these mechanistic links. From a clinical perspective, AACV could be integrated into existing workflows with minimal effort, as it can be derived from routine CT scans. The semi-automated measurement is fast, cost-effective, and requires no additional imaging, making it feasible for broad implementation.

The present analysis has several limitations to address. First, retrospective single-centre studies are prone to possible inherent bias. Moreover, detailed information on important cardiovascular risk factors such as smoking status, body mass index, diabetes, hypertension, and cardiovascular history was not available for all patients and could therefore not be incorporated into the multivariable models, which may have influenced both AACV and survival. In particular, AACV may serve as an imaging-derived surrogate marker of overall cardiovascular frailty and general health status, which could influence treatment tolerance, postoperative recovery, and long-term survival. An additional limitation is the absence of data on the cause of death, preventing differentiation between cancer-related and non-cancer-related mortality. Therefore, an influence of cardiovascular comorbidity on overall survival cannot be excluded, although the consistent association with disease-free survival supports a potential oncologic relevance of AACV. Another limitation is that the cohort was derived from a surgical database and therefore includes only patients who underwent curative-intent esophagectomy. Patients who became ineligible for surgery after neoadjuvant therapy, were not captured, which may introduce selection bias. Furthermore, surgical techniques evolved during the study period, including increasing adoption of minimally invasive and robotic-assisted esophagectomy, which may represent a potential confounding factor.

In conclusion, CT-quantified abdominal aortic calcification provides independent prognostic information in patients with esophageal cancer undergoing neoadjuvant therapy followed by curative resection.

## Supplementary Information

Below is the link to the electronic supplementary material.

Supplementary Material 1

## Data Availability

The datasets used and analyzed during the current study are available from the corresponding author on reasonable request.

## Abbreviations

AACV: Abdominal aortic calcification volume
AAIACV: Combined abdominal aortic and iliac artery calcification volume
CAC: Coronary artery calcification
CI: Confidence interval
CROSS: Chemoradiotherapy for oesophageal cancer followed by surgery study
CT: Computed tomography
DICOM: Digital imaging and communications in medicine
EC: Esophageal cancer
FLOT: 5–fluorouracil, leucovorin, oxaliplatin, docetaxel
HR: Hazard ratio
HU: Hounsfield units
IACV: Iliac artery calcification volume
ICC: Intraclass correlation coefficient
IQR: Interquartile range
OS: Overall survival
PACS: Picture archiving and communication system
ROI: Region of interest
SPSS: Statistical package for the social sciences
TNM: Tumor–node–metastasis
