# Supplementary material for: Prognostic value of abdominal aortic calcification in patients with esophageal cancer undergoing neoadjuvant therapy and esophagectomy
Source: Abdom Radiol (NY). 2026 Apr 21;51(11):5579–88. doi: 10.1007/s00261-026-05511-3 (PMC13672523; doi:10.1007/s00261-026-05511-3)
Supplement: Supplementary file 1 [file 261_2026_5511_MOESM1_ESM.docx]

**Supplementary Material 1.** Restricted cubic spline analysis of abdominal aortic calcification volume (AACV) and survival outcomes. (a) Overall survival (OS) and (b) disease-free survival (DFS). The solid line represents the estimated hazard ratio across AACV values, with shaded areas indicating 95% confidence intervals. The vertical dashed line marks the median AACV used as the reference (HR = 1). No evidence of non-linearity was observed (OS: p = 0.61; DFS: p = 0.67).
